# A Practical Treatise on the Diseases of the Eye

**Published:** 1821-03-01

**Authors:** 


*A practical Treatise on the Diseases of the Eye.* By John Vetch, M.D. F.R.S. E. Member of the Royal Medical Society of Edinburgh, and the Medico-Chirurgical Society of London; lately Physician to the Forces, and principal Medical Officer to the Ophthalmia Military Hospital. Three plates, 268 pages. London, November, 1820.

The early authors of the Greek School, the Romans and the Asiatic Arabians, considered ophthalmic diseases as a constituent part of medical science, and the accuracy and minuteness of their descriptions testify their knowledge of all that pertains to them. Celsus, indeed, was of opinion that the same person might excel in the practice of both branches of the profession; and he considered him most deserving of praise, who understood the greatest number of diseases.

"Ego eundem quidem hominem posse omnia ista præstare concipio: atque, ubi se diviserunt, eum laudo, qui quamplurimum percipit." *A. C. Celsus de Medicinâ, p.* 406.

It was not, however, until the final erection of surgery into a separate department, that we find diseases of the eyes selected as a distinct branch of medical education. This appears to have commenced under the Arabians of Spain; and in Germany and Italy the science of ophthalmology has continued to constitute a part of medical instruction. To these countries we are indebted for a great share of the impulse now given to the subject here; but our thanks are chiefly due to Mr. Wardrop and the late Mr. Saunders, for originating attempts to blend the knowledge and practice of the oculist with the chirurgical art. Other surgeons are now taking up the subject, in imitation of the continental practitioners, charitable institutions are formed and forming for the relief of those afflicted with diseases of the eye, and in a short time we shall become as eminent in the knowledge of ophthalmic as of other branches of surgery.

To a work so strictly practical, the result of long and extensive experience, analysis, of course, is peculiarly applicable, and we hope, by the following *precis* of its contents, to shew good cause why every practitioner, who has the means, should possess himself of so valuable a contribution to ophthalmic science. Our readers will therefore remember that, though not always speaking the language, we are always delivering the sentiments of our experienced author, in the following analytical delineation.

The work before us opens with observations on the *general character and treatment of ophthalmic inflammation.*

The conjunctiva is little else than a congeries of lymphatic veins, leading from the circumference of the cornea to the duplicature of the membrane, by which the serous portion of the blood is returned from the internal parts of the eye into the general circulation. Hence inflammation of this membrane partakes more of a venous, than arterial action. The sclerotic coat, on the contrary, being supplied with blood from the ophthalmic artery, a branch of the internal carotid, when inflamed, exhibits all the marks of strict arterial action.

Inflammation and its consequences do not take place in the cornea, until inflammatory action has been set up in the vessels of the sclerotic coat, even when the exciting cause is applied directly to the substance of the cornea.

Iritis, or inflammation of the iris, is to be considered as a distinct form of sclerotic inflammation, connected with some idiosyncracy, or morbid diathesis, previously existing in the constitution. Before any degree of acute inflammation can establish itself either in the cornea or in the iris, a similar action must have taken place in the sclerotic coat.

Inflammation of the sclerotic coat is slow in its progress. It appears at the circumference of the cornea, forming a zone around it. Intolerance of light attends it. These circumstances distinguish this from conjunctival ophthalmy. Other peculiarities are also present in the former, when arterial action runs high: as the discharge of albuminous matter and the formation of chemosis. In the advanced stage of sclerotic inflammation pus is discharged, having a specific property.

In the treatment of conjunctive inflammation, when venesection is employed, it must be carried to the extent of producing syncope, by opening a large vein on the arm or neck. A sudden collapse of the vessels is thus produced, which will often prove the cure of the disease, as we have repeatedly seen. This salutary effect of syncope can only be ascribed to the laxity of the vessels, which renders them unable to recover their former excitement.

Local bleeding by cupping and leeches has generally sufficient control over sclerotic inflammation, to render any larger discharge of blood unnecessary. An advantageous situation for the application of leeches is the conjunctival lining of the inferior palpebra, and great benefit may be derived from applying them to the septum nasi.

The exclusion of light is often more detrimental than useful, particularly by confining heated air, and preventing the access of a more temperate atmosphere.

All washes and eye-glasses are for the most part worse than useless. The only proper mode of directing such applications is to inject the fluid over the whole surface of the extended eye-lids by an elastic gum-syringe.

Blisters, when applied to the temples, never fail to prove injurious, by increasing the action of the arterial trunks, over which they are placed. In cases of purulent ophthalmy, Dr. Vetch has observed some relief from the application of blisters to the external surface of the palpebræ.

While combating the force of the general inflammation, the important structure of the iris may be secured from injury by the employment of narcotics; as hyoscyamus, belladonna, and stramonium. These may also be used as a mechanical force in detaching any recent adhesions. The argenti nitras may often be applied as an excellent sedative.

For the purpose of altering the violent and purulent state of the conjunctive membrane, it is impossible to possess a medicine of greater efficacy than the liquor plumbi sub acetatis, infused in an undiluted state. Nicotiana, as a narcotic and astringent, applied externally, is of singular use in abating both the pain and the excessive tumefaction.

Inflammation is never equally advanced or equally urgent in both eyes at the same time. Our remedial measures must be as vigorous in the one instance as in the other.

The following is a tabular view of the different forms of ophthalmic inflammation, consonant with Dr. Vetch's outline:—

OPHTHALMIA, OR CONJUNCTIVAL INFLAMMATION.

*Species* I. Catarrhal ophthalmia, or *ophthalmia mitior*. Sporadic, endemic and epidemic; with or without chemosis.

*Species* 2. Oph. purulenta, or puriform ophthalmy. *Oph. gravior, the lippitudo, oph. vera, oph. humida* of the ancients. *Blephoroblenorrhœa* and *ophthalmoblenorrhœa* of the Germans.

Var. *a.* Ophthalmia of Infants.
*b.* ———— produced by the infection of ophthalmic virus.
*c.* ———— by the infection of gonorrhœal virus.
*d.* ———— by the metastasis of gonorrhœal inflammation.
*e.* ———— rheumatic, syphilitic and arthritic.

OPHTHALMITIS SCLEROTICA.

*Species* 1. Idiopathic, or corneal.
2. Irridial, or symptomatic.

We next come to the important subjects of *ophthalmitis sclerotica; ulceration of the cornea; and opaque cornea.*

The causes of sclerotic ophthalmy are external or internal, general or specific. When it appears after the decline of exanthematous disorders, which are among the internal causes, it has been distinguished by the name of morbillous and variolous ophthalmia. Dr. Vetch divides the sclerotic ophthalmitis into *sclerotico-corneal inflammation* and *sclerotico-choroidæal inflammation*. In the former the exciting cause is mostly external, and the inflammation occupies the external surface; in the latter, the inflammation manifests a rheumatic character, and by its ready extension inwards to the iris is chiefly known as iritis.

The first set of symptoms of the *sclerotic inflammation* are of a generic nature: increased vascularity, morbid sensibility to the impression of light, contraction of the pupil, pain, heat, augmented secretion of the lachrymal fluid, pyrexia.

The vessels, diverging as they come forwards, produce a zone of minute, hair-like vessels, distinguished by their rectilinear direction and their uniform concentration towards the margin of the cornea. Their colour advances from that of a delicate pink, or damask rose, to a deeper hue, and imparts a faint blush to the part immediately surrounding it.

As yet the back part of the eye and the surface of the palpebræ preserve their natural appearance.

When the vessels encroach on the margin of the cornea photophobia (intolerance of light) immediately occurs. In this situation they are considerably darker in colour, and give the cornea a relaxed appearance; and after the inflammation has abated, a loss of transparency is liable to remain. In old age this opacity of the circumference of the cornea often forms a complete ring, and is called annulus senilis.

Photophobia and contraction of the pupil, unless when accompanied with more evident signs of iritis, are merely symptomatic of the inflammation of the sclerotica and cornea. When the pupil is much contracted, the lighter or greener shade towards its inner circumference, appears to be the effect of a greater expansion of its fibres. We have known this appearance of the iris deter some surgeons from endeavouring to remove the disease; the sight being considered irrecoverable.

The inflammatory action of the vessels of the cornea has sometimes the effect of depositing lymph in the middle lamina, which has the appearance of pus. It is only in colour that it resembles pus, for it possesses no fluidity.

### *Treatment of Sclerotic Inflammation.*

Our first object is to ascertain the presence of any foreign matter. This may be done by everting the upper eyelid with a blunt probe, or by injecting tepid water or infusion of poppy or mallow over the everted membrane and eye itself, with an elastic gum-syringe.

At all periods of the inflammation Dr. Vetch has found the abstraction of blood from the temple, by means of cupping, to be the best method of subduing the disease. This operation may be aided by the application of leeches. General bleeding may sometimes be requisite, and antimonials in nauseating doses. Temporal arteriotomy has lately been objected to, and our author remarks that every advantage may be more safely and more effectually attained by venesection or cupping.

In violent inflammation, where photophoby is urgent, great assistance is gained by the constant application of a solution of opium or hyoscyamus, of a temperature below that of the atmosphere. In slighter attacks more relief will be afforded by the occasional use of hot fomentations, or of the vapour of water and vinegar. So obvious is the tendency of warm poultices and long continued fomentations to effect relief, by accelerating the destruction of the cornea, that Dr. Vetch says,

"I should consider any patient entitled to recover damages, in whom the disease has terminated unfavourably, whenever it has done so under the application of a poultice." P. 40.

Argenti nitras appears to produce some specific change in the state of the vessels of the cornea, and when properly used, gives no pain. The vessels leading into the cornea should be barely touched with the caustic formed into a finely pointed pencil. After this let the eye be immediately washed by injecting a little tepid water with an elastic gum-syringe. A solution of the nitrate of silver in different proportions may supply the place of all the other metallic salts; and a convenient vehicle is made with mucilage and oleum ricini for the exhibition of vin. opii, extr. hyoscyami and liq. plumbi subacet.

Dr. Vetch's testimony respecting the evacuation of the aqueous humour, proposed by Mr. Wardrop, goes more to establish the safety than the expediency of the operation.

On the least affection of the iris being perceived, the extract of hyoscyamus should be employed, together with the means of subduing the external inflammation. Other vegetables besides those in use have the property of influencing the action of the iris: as euphrasia and anagallis arvensis.

After the violence of the disease has been subdued, photophobia with lachrymation and quick pulse may be relieved by digitalis internally administered.

The strictest attention must be paid to diet, and occasional purgatives will be required.

*Ulceration of the Cornea.*

When this takes place in consequence of purulent ophthalmy, it commences on the external surface, and gradually deepens and enlarges, till it penetrates the whole thickness of the part. When it is the effect of primary sclerotic ophthalmia, it begins by *apparent* apostemation in the substance of the cornea, whence ulceration proceeds either inwards, so as to terminate in hypopion, or in an external ulcer. In the external tunic of the cornea, ulceration may have the appearance of excoriation or abrasion. Sometimes a papula is observed communicating with a plexus of vessels on the conjunctiva. Small circular herpetic excoriations not unfrequently appear, combined with sub-acute, sclerotic inflammation, and tinea of the tarsi. A hard, white, shining, ragged, irregular crust, is sometimes formed on the denuded surface of the middle lamina. It is long stationary, prevents the march of ulceration into the middle lamina, and leaves no opacity behind; an event which always happens when the middle lamina is the seat of ulceration. When the crust is absorbed by the progress of ulceration, a dusky yellowness, arising from a deposition of lymph, is sometimes discernible beneath. The removal of this crust by artificial means is extremely injudicious.

The yellowish substance found between the laminæ of the cornea, having as we before observed the appearance of pus and the nature and properties of lymph, should be removed. If suffered to remain, leucoma will follow. It may be removed in a mass on the point of a lancet or couching needle, or wound round the point of the instrument, if it should have a communication with the side or edge of the ulcer. A blueish appearance follows, from which the part gradually recovers. When all the lymph cannot be removed, slight scarifications assist in throwing it off, and occasion the reproduction of transparent cornea. To prevent a laceration of the serous tunic, the blade of the instrument should be applied almost parallel with the slough.

The moment the ulcer penetrates the inner coat, the aqueous humour escapes; and if the ulcer is within the line of the iris, an immediate approximation of the latter to the opening takes place, constituting procidentia iridis. After the adhesive process has taken place, the inner membrane projects in the form of a vesicle, and when this occupies a large portion of the cornea, it is called staphyloma. The origin and progress of this affection, we believe, has never been properly described by any writer before Dr. Vetch. Its resemblance to the head of the mouse-fly has obtained for it the name of myocephalon. The vesicle should not, if possible, be ruptured. Its formation may be prevented by puncturing the cornea at a place as remote as possible from the ulcer. Scarification of the slough, and the application to the ulcer of a solution of nitrate of silver, infusion of nicotiana, or calomel, may be employed occasionally with advantage. To the incipient staphyloma caustic should be applied, and it will be sufficient that it barely and instantaneously touches the surface. The iris will thus be saved from any permanent adhesion. Scarpa, believing ulcer as the cause of ophthalmia directs the caustic to be applied, till a slough is produced; but Dr. Vetch's experience leads him to suppose, that such a severe practice would be fatal to the success of the remedy.

The colour of the tumour, when staphyloma is complete, is uniformly blue. When the pain of distention is great, particularly in damp weather, the evacuation of the aqueous humour is often required. The tumour having acquired its full growth, white bands traverse it; but the interstices retain their original tenuity and blueness. These bands are thick, hard, and bleed on being cut, and gradually extend over the whole tumour. While this process is going on, the repeated rupturing of the thin parts diminishes the tumour, which finally subsides, leaving an indelible leucoma over the place which it occupied. An artificial pupil may then be formed. Dr. Vetch objects to the removal of the apex, recommended by Scarpa, and has employed, with success, caustic and the introduction of a seton, in order to accomplish the gradual diminution of the tumour.

The cornea is liable to assume a conical shape without any evident alteration of structure. With a view to improve vision, the aqueous humour has been evacuated, the lens removed and pressure long continued, to no purpose.

A pellucid dimple, supposed to proceed from intersticial absorption, is liable to appear on the cornea. It is generally stationary and free from danger; but Dr. V. has seen it, in many cases, become an open ulcer, terminating in procidentia iridis, after having been preceded by inflammation in the sclerotica.

*Opaque Cornea.*

After the cessation of conjunctival ophthalmia, the villous elongation of the vessels of the palpebral linings often acquire a farther increase of size, and produce a granulated surface, with a secretion of pus. An inflammatory state of the sclerotic vessels gradually follows, the superficial vessels become varicose, the conjunctiva assumes a dusky and loaded appearance, and the cornea throughout becomes opaque. This opacity of the cornea is generally attended with dark coloured vessels, increasing as they come outwards, and being distinctly varicose. By degrees, the cornea becomes softened, and at length, small sloughs appear on its surface.

This disease seems to arise from a loss of balance in the supplying and returning vessels of the cornea. The diseased structure bleeds profusely, when cut into, and there is a general oozing of pus.

This state of the linings of the palpebræ, termed by the Greek writers trachoma and sycosis, was distinctly described by all ancient authors; but the knowledge of it appears to have been lost until the time of St. Ives, who performed, a hundred years ago, the same kind of service to surgery, which Mr. Saunders lately rendered it.

In the treatment of this disease, Dr. Vetch is decidedly, and we think justly, averse to excision.

"The connexion of the disease of the cornea with that of the lids, and the eversion of the latter for treatment or examination, was practised by Mr. Saunders, who taught it to Sir W. Adams, and was resorted to by myself without any knowledge of the practice of Mr. Saunders, or the existence of Sir W. Adams, though I did not then; nor do I at this moment, consider the eversion of the eyelid to be generally necessary to the cure of the disease, its frequent repetition has appeared to me, not only unnecessary but prejudicial; and I have met with more success by simply raising it with the thumb of the left hand, so as to admit the application of a small porte-crayon armed with the blue stone or nitrate of silver, than I ever did by the complete eversion of the palpebræ; nor will I yield the evidence of my own sense and experience on this point, to any conjectural reasoning whatever. The treatment of this complaint forms but one feature of a disease, which, at the time that I met with it, *was new to the profession in this country*, and although the practice proved *successful to the utmost possible degree, it has been condemned without inquiry* and without appeal, and *in a quarter where professional discussions cannot be entertained.*

"The whole power of high and official patronage has been employed to ensure the success of the operation for removing the granulation of the palpebræ by the knife; the failures have been concealed by every possible subterfuge; Nature, however, has proved her superiority, and it is now generally known that the operation, while it has been highly injurious in some cases, *has only proved successful when aided by those applications, which it was introduced to supercede*, and this success has been so limited, as to prove that their use is not yet properly understood, where the greatest pretensions have been advanced." P. 78, 79, 80.

Blood should be abstracted from the temples by cupping, and when the formation of a slough is apprehended, a leech should be applied to the inner surface of the lower eyelid. Determination of blood to the head should be avoided, and great limitation of diet enforced.

The escharotics abovementioned, being pointed, and fixed in a porte-crayon, are to be applied with great delicacy, and in so many points only, as will produce a gradual change in the condition of the part, without the sloughing process. The eyelid seldom requires to be everted. As long as any purulency remains, this plan will be much aided by the daily use of the *liq. plumbi subacet.* When the disease resists these remedies, and the surface becomes hard and warty, Dr. V. has had recourse to a fine powder of ærugo or alumen ust. applied to the everted surface. These substances must be introduced in minute quantities with a camel's hair-pencil and carefully washed off, before the eyelid is returned. The kali purum may be substituted for the other caustics in these obstinate cases.

The 3d chapter of Dr. Vetch's work is on *ophthalmitis iritica vel sclerotica interna.*

This is a sub-acute ophthalmy, and may be referred to the general character of rheumatic inflammation, as opposed to the phlegmonic form of it. The former has a tendency to injure the structure of the iris and adjoining parts; the latter that of the cornea. The sclerotica interna seldom goes beyond the limits of adhesive formation, and this process may originate in the iris, while the redness in the sclerotic coat appears secondary.

The most frequent cause of this form of sclerotic inflammation, is the translation of rheumatism and gout. The pre-disposition may arise from the similarity of structure in the sclerotic coat and the tendinous expansions. Inflammation of this coat, with a disposition to attack the iris, is a frequent occurrence while the system is under the influence of mercury, and the specific efficacy of mercury, in checking the same form of inflammation, being an ascertained fact, there is some difficulty in explaining two such opposite effects. Other parts composed of the white, fibrous tissue are subject to the same effects of mercury. This medicine has the property of increasing the capillary circulation, and, while it is thus operating, if any part be peculiarly exposed to the influence of cold, there is danger of the increased activity of the vessels being suppressed. Then rheumatic inflammation follows. This may happen to the larger joints, to the muscular fibres of any part of the alimentary canal, and we have known it occur in the tendinous expansion of the occipito-frontalis muscle. From analogy, we therefore agree with Dr. Vetch in considering the sclerotic inflammation, occurring during the use of mercury, as a rheumatic inflammation, occasioned by an excited circulation in the capillary system being suddenly suppressed by the application of cold.

Ophthalmitis iritica presents the zone and other general appearances we have already described. The pain is of a stabbing or lacerating kind; is increased by warmth, and accompanied by epiphora. Photophobia is not intense, and, as the disease penetrates the iris, it sensibly diminishes. The patient perceives sudden flashes of ignited bodies; and, in bad cases, the sclerotica, losing its spherical form, suffers a partial projection, denominated *staphyloma sclerotica.* The pupil for some time preserves its circular shape; the smaller ring of the iris becomes of a light red colour; the greater ring takes a greenish hue; as the pupil contracts, its margin shews some little irregularities; the pupil itself loses its shining, black appearance; and the inflammation having extended to the capsule of the lens, vision becomes sensibly impaired. The iris swells and presents a convex surface towards the cornea. The pupil becoming more angular, a gray, ash-coloured membrane appears to come forward from behind the border of the iris, which forms a medium of adhesion between its pupillary margin and the capsule of the lens. The sight is more impaired, and at last the eye becomes insensible to light. As the iris approaches, the cornea loses its healthy aspect. At length, one or more small tumours of a dark orange-colour appear on the surface of the iris, which terminate in suppuration. The matter which they contain, falling into the anterior chamber, produces hypopion, and the remains of the cyst are seen floating in the aqueous humour. The inflammation generally terminates in the effects which belong to the adhesive stage. The formation of the adhesive membrane produces the dilated, contracted, and obliterated pupil. A permanent dilatation may be owing to the adhesion of the greater ring of the iris, or to some disease in the choroid coat, or of the membrane lately described by Dr. Jacob, which lies between it and the retina. The pupil is frequently dragged, more or less, from the centre of the eye. The lymphatic membrane, spreading itself over the capsule of the lens, constitutes an important variety of false cataract, which, when viewed through a magnifying glass, appears to be vascular, and terminates in true capsular or lenticular cataract.

When iritis appears during the progress of syphilis, the iris becomes less moveable, the pupil contracts and lies towards the inner angle; the lens and greater ring change colour; the pupil becomes angular, and the iris advances. Photophobia increasing in the evening, and a fixed pain, chiefly in the eyebrow, attend. As the morning approaches, the patient enjoys some repose. Small red, or orange-coluored tumours appear at the pupilary or ciliary margin, and suppurate.

Iritis, with a primary inflammation of the sclerotic coat, is not a very unfrequent effect of gout in irritable subjects. With other premonitory symptoms, the sclerotic appears reddened, and does not propagate its inflammation to the border of the cornea, but, according to the observation of Professor Beer, leaves a narrow, blueish white ring around its edge, which can only be observed in the early stage of the attack; for the space is filled up by the vessels of the conjunctiva, which become varicose. When the sclerotic inflammation is formed, the common appearance of iritis comes on, but the pupil remains in its natural situation. The lymphatic membrane spreads rapidly, and the pupil becomes narrower, and at length closes. Sometimes the lens forms into a green cataract.

*Treatment.* Repeated cupping in the temples, as long as inflammation may appear in the sclerotic or iris, is necessary for the cure of all these species, and insures the efficacy of other means. A small quantity of mercurial ointment with opium, rubbed into the eyelid and temple night and morning, seems to have a specific agency in arresting the progress of the disease in the iris; the same may be said of the local use of stramonium and hyoscyamus. If the disease have occurred during a course of mercury, we may renew its exhibition with safety and advantage. When gastric derangement appears to be connected with rheumatic inflammation, we must try to remove that derangement. In protracted cases of this kind, the operation of an emetic often cuts short the disease in the eye. Dr. Vetch speaks highly of the free use of digitalis, as a powerful auxiliary; and we have repeatedly seen the best effects from a seton in the neck.

Chapter IV. is on lenticular and capsular inflammation.

Until very lately, little has been added to our knowledge of the cataract since the days of Celsus and Galen.

The lens is subject to an acute inflammation, which may be called *lentitis.* Cataract exemplifies the adhesive; this the suppurative process. In lentitis, the lens gives out a ropy matter in extreme cases, which, after traversing the anterior chamber, reaches the external surface through the cornea, without losing its continuity with the lens. The escape of the aqueous humour is, in great measure, prevented by this ropy filament. Lentitis, in its common form, does not proceed beyond the adhesive stage.

Cataract is of two kinds: lenticular and capsular. It is hereditary. The lenticular cataract generally exhibits a dark gray colour, with a tinge of yellow, and remains darker at the centre, than at the border of the pupil, even when the whole has become opaque This disease is slow, has no influence on the iris, and does not altogether destroy vision. The space of the posterior chamber is distinctly to be seen, and the cloudiness is convex and without spots.

Capsular cataract begins with a few white, shining streaks or spots; its colour is never *uniformly* deep; and it affects the motions of the iris. Opacity of the lens usually soon follows. Capsular cataract may be confined to the anterior or posterior hemisphere, or both may be affected; but the capsulo-lenticular is the most frequent form of the disease, of which there are many varieties. The cataracta capsulo-lenticularis centralis in the human subject, is said by Beer to be observable soon after birth, and, sometimes, to remain unaltered during the whole period of life. In the horse, this central speck has an almost uniform tendency to terminate in capsulo-lenticular cataract. We may here take the opportunity of observing that, a white spot is to be seen in a sound eye of this animal, by making the rays of light fall in a particular direction; as by turning his head slightly round in the stall opposite the light.

The cataracta caps. lentic. siliquata, is the cataract of infants. It has a light gray colour, and is seen at a considerable distance from the pupil. This recession of the lens is owing to interstitial absorption, which brings the hemispheres of the crystaloida nearly into contact.

*False cataract* is of two kinds: the one arising from inflammation, which throws out a pseudo-membrane between the uvea and the lens; and the other proceeding from violent concussion, by which a portion of the tapetum of the uvea is detached, and rests itself upon the anterior capsule.

It is of importance to distinguish the consistence of a cataract. *Hard cataract* is mostly of a dark colour and distant; and the iris is free, and, when expanded, vision is tolerably distinct. *Black cataract* resembles amaurosis, but is ascertained by dilating the pupil. *Fluid cataract* is always joined with perfect opacity of the capsule. *Congenital cataract* is always fluid at first. The size of the fluid cataract is generally larger than the natural lens.

Dr. Vetch's account of the complications of cataract is short, but useful and explicit.

Application to microscopic objects predisposes to cataract, which forms, according to our author, in the eye which is kept shut. Sudden and strong light, especially in infants, produces inflammation ending in permanent, central opacity, or in cataract. Exposure of the head and eyes to the heat and light of fires has the effect of predisposing to cataract. The fumes arising from the oxydation of metals, dust, the abuse of spirits, predispose to the disease in elderly persons.

*Treatment.* Generally speaking, the same line of treatment, which has been recommended for iritis, is equally applicable to the early stage of capsular inflammation, and it will be much assisted, as we have found from experience, by the use of setons or issues and rubifacients.

When the operation is requisite, especially if done by absorption, there is no advantage in waiting for the more perfect formation of the cataract, which is often capable of being removed at any stage by breaking up the lens; unless other circumstances should render delay adviseable.

The sixth chapter is on *Amaurosis.*

An interrupted vision is one of the first symptoms of this disease. Myopia, presbyopia, diplopia, and strabismus, are also associated with it. Light is sometimes sought for; at others it is painful. When amaurotic blindness is complete, the eye is insensible to light; and this may happen in one or both eyes. Amaurotic amblyopia is sometimes periodical, especially in hysteria and chlorosis. Total blindness is apt to take place in the end. Amaurosis appears in nyctalopia and hemeralopia. The former occurs to horses as well as to the human species; and the latter is sometimes epidemic. Whether the pupil be dilated or contracted, it no longer obeys the stimulus of light. The pupilary border becomes more or less angular, and the opening looks less clear than that of a sound eye. During the formation of the disease, pain in the eye or in the head, giddiness, and other symptoms of cerebral affection, are present. The middle period of life, and dark eyes, are most liable to amaurosis; and it is distinctly hereditary. Its production is favoured by continued observation of microscopic objects, and by any cause capable of augmenting the quantity of blood in the vessels of the head; by lightning; by violent mental emotions; by a disordered state of the abdominal viscera; by narcotics; by constitutional idiosyncracy with respect to diet, &c. by the internal use of lead. One form of amaurosis appears to be owing to a deficiency of pigment, and rarely admits of any cure, although it seldom proceeds to absolute blindness. The bottom of the eye appears pale, yellow, and glittering; and when the branches of the central artery become visible, and the iris pale, the eye resembles that of the cat. This species of the disease is met with in old people, and in younger persons after continued fever, in phthisis, and in atrophy.

Amaurotic amblyopia is seldom perfectly cured, excepting when it arises from narcotics. The action of the iris affords no sure criterion respecting the condition of the retina; and when one eye is attacked without any perceptible cause in the organ itself, the other may be expected to become diseased.

*Treatment.* Bleeding must be carried to the extent of producing syncope; leeches, purgatives, antimonial emetics, pediluvium, seclusion from the light, cold applications, counter-irritants to the neck and behind the ear, sinapisms to the feet and legs; the reproduction of habitual discharge or cutaneous disease, must also be had recourse to. Dr. V. says, the most advantageous situation for the leeches is the septum nasi. He strongly reprobates the indiscriminate use of Galvanism and electricity.

The second division of our excellent author's work commences with the subject of *conjunctival inflammation;* and first on *catarrhal inflammation, arising from climate and atmospherical changes.*

Dr. Vetch's observations agree with the testimony of most writers, that a humid state of the atmosphere, combined with a certain degree of heat, is that which proves most productive of ophthalmia.

### *Catarrhal Ophthalmia.*

A catarrhal inflammation of the conjunctiva is seldom met with in England; but Dr. V. has reason to believe that it is by no means unfrequent in Ireland.

The causes of epidemic ophthalmia in northern countries seem to have a less extensive influence than those which favour the production of epidemic catarrh or influenza. The influenza sometimes prevails over whole kingdoms, while epidemic ophthalmia is generally confined to particular districts. Hence Dr. V. is disposed to believe that the latter disease is more frequently conveyed by infection, than generated. At the same time he remarks, that the excessive crowding together of men, as in barracks, will often of itself engender inflammation of the conjunctiva; and render the constitution more disposed to be acted upon by the exciting cause whether natural or engendered.

The epidemic ophthalmy rarely takes on the purulent action. It commences with stiffness of the eyelids, fulness of the eye, sense of grittiness on the conjunctiva, pricking pain in the lachrymal caruncle. There is either aridity or copious lachrymal discharge, attended with scalding, and mixed with mucus. The lining of the eyelid appears of a mottled, red colour. The conjunctiva is generally covered with patches of large vessels, and the chemosis is transparent and often partial. About the fifth day these symptoms decline, and the mucous discharge increases. In some cases suppurative inflammation follows, imparting to the conjunctiva a fleshy and villous appearance, with a greater degree of chemosis, and with distention of the cornea.

*Treatment.* Stimulants put a stop to the progress of this inflammation, by carrying the excitement of the vessels beyond the action of the disease. Spirits, vinegar, snuff, &c. when applied externally, have this effect; and a stimulating ointment at night, with the daily use of a solution of sulphate of zinc, are in most cases sufficient. In individual instances of catarrhal ophthalmy, a violent inflammation without purulency will often require a decisive use of the lancet.

We now come to an important and melancholy subject, the *purulent ophthalmia of the British army.*

When this disease prevails extensively, two forms of it are produced; the one from the impression of atmospherical causes, and the other from infection. Dr. Vetch informs us that the puriform discharge from the conjunctiva, arising from whatever causes, operates as an animal virus, when applied to a healthy eye; and the disease proceeding from this source is by far the more violent and malignant of the two. The continuance of purulent ophthalmy in the English and French armies after their return home, and the repeated occurrence of it in the troops of this country, are striking proofs of its contagious nature. Dr. Edmonstone first made the public acquainted with its contagious nature, and in 1807 Dr. Vetch first established the fact, that the disease, as it appeared in this country, was *exclusively* produced by the application of the morbific secretion. A humid atmosphere has the effect of aggravating the symptoms; and for months or years after an attack, the eye is found to be influenced by atmospherical vicissitudes.

Our author presents us with a general view of the introduction, progress, and decline of the purulent ophthalmia in the British army; from which it appears that the disease was kept up by confining the soldiers to barracks, many of them in marshy situations, and by a new and fatiguing system of drill, which was then in full operation. On the contrary,

"The French army, after its return from Egypt, instead of being confined to barracks, and harrassed by a fastidious discipline, proceeded from conquest to conquest, bivouacking in the field, or quartered on the inhabitants of the countries, which they subdued. The means therefore of disseminating the infection, if it existed, did not occur to the same extent." P. 187.

The smallest quantity of virus will produce the disease which sometimes follows in less than twelve hours; and the same person may again receive the infection. Both the eyes are generally attacked. A mottled appearance, and then a fleshy redness are observable in the lining of the lower eyelid. From this situation the inflammation spreads rapidly over the whole of the conjunctive membrane, as far as the cornea. This is seldom accompanied with any other sensation than a stiffness, or such a feeling as would arise from sand rolling in the eye. These sensations proceed from the turgid vessels. Chemosis, bounded by the cornea, more or less complete, succeeds, together with œdema of the palpebræ, and inversion of the eyelids. Pus now flows in a continued stream, and attacks of severe pain commence in the eye. The pain is often intermittent and migratory, and sooner or later is succeeded by a sense of rupture of the cornea, and a gush of scalding water. The pulse is unaffected, unless the lancet have been freely employed; and the health is unimpaired; but sleep is absent. At length the external tumefaction subsides, and a gaping appearance of the eye presents itself. The cilia are everted and distant, and the eyelids cannot reassume their natural state in consequence of the granulated state of the conjunctiva, which lines them, and which is protruded more or less. The fluid discharged is the aqueous humour, which is secreted in great abundance. The ulcer in the cornea is sometimes followed by staphyloma; and in some cases the lens has presented itself at the orifice; but as soon as the capsule has given way, the lens and part of the vitreous humour have escaped. This destruction of the globe of the eye for the most part, as in horses, insures the safety of the other. When one eye is lost by staphyloma, and the other remains useful, it is well to lay the sac open and extract the lens.

*Treatment.* The liquor plumbi subacet. in its undiluted state is the application which Dr. Vetch recommends as the most efficacious. Its employment is followed by a sensation resembling that which arises from sand in the eye, and by copious lachrymation.

Venesection is another powerful expedient. In our general remarks on ophthalmy, we pointed out the necessity of inducing syncope, when the conjunctiva is the seat of inflammation; and have infinite satisfaction in confirming Dr. Vetch's testimony of the excellence of this practice, which we have found invariably successful. It is not sufficient that the vessels become pale: an actual deliquium animi must be produced, otherwise the vessels will recover their former activity, and the disease advance.

A free exposure to, and frequent change of, air are serviceable.

After Dr. V. had published his former work, he was induced to try the effect of various narcotics in this disease; and found that nicotiana possessed the properties of acting as an astringent, and of diminishing the œdema of the palpebræ. It has also the effect of relieving the pain and perpetual watchfulness. It should be applied at bed-time in form of infusion, in the proportion of two drachms to eight ounces of water. An infusion of galls and opium may be substituted for the former.

When active measures have been neglected in the beginning, it may be advantageous to employ local bleeding, which must be regulated in great measure by the pain; and, when the discharge continues acrid, blisters on the neck and behind the ears prove serviceable.

For the purpose of supporting nausea, Dr. V. employs an infusion of nicotiana. This, however, it must be recollected, is only a measure of secondary importance.

Where venesection has failed to produce syncope, and the pain indicates that distension is going on; the external swelling being so far reduced, as to expose the cornea; the operation of puncturing the cornea may be had recourse to, and repeated, if necessary. A frequent repetition of this operation, it should be remembered, generally occasions a partial adhesion of the iris.

As long as the lower eyelid has internally a red and villous appearance, there will be danger of a relapse, from exposure to cold or other exciting causes.

*Eversion of the eyelids, or ectropion,* following Egyptian ophthalmia, is attended with great tumefaction and a granulated state of the protruded part. It is occasioned by the action of the orbicularis muscle, after the external œdema of the eyelids begins to subside. The tumour is the effect of strangulation, and the hypersarcosis belongs only to the surface. Instead of cutting away the granulated surface day after day, as was formerly the practice, the everted portion is to be exposed to a light and careful application of argenti nitras, and to be returned and secured in its place with a compress and straps of plaster or bandage. Every time the eye is cleaned, this process is to be repeated; and Dr. V. says,

> "In the course of a few days the tendency to protrude will disappear, and at the end of a fortnight the patient will have so far recovered the use of the muscles of the part, as to be able, by their means alone, to raise open the eyelids at pleasure." P. 229.

*Gonorrhœal Ophthalmia.*

Of this disease there appear to be two varieties: one depending upon a constitutional communication of the diseased action in the urethra, and another arising from the application of the gonorrhœal virus. The inflammation in the former is of the rheumatic kind. In external appearance it differs chiefly from the other, by the œdema being principally confined to the conjunctiva, where it is attached to the eye, producing chemosis; so that the eye is seldom concealed. The discharge is less, is of a more viscid consistence, and deeper yellow colour. There is more photophobia, and a greater tendency to attack the internal parts, and to destroy the cornea with slough or ulceration. In the treatment of this form of the disease, the free use of cold applications is best suited to promote a cure. Poultices are highly improper, and general bleeding is not so efficacious as in those cases proceeding from inoculation.

The treatment of gonorrhœal ophthalmia, arising from the latter source, may be successfully conducted on the same plan, which has been laid down for the cure of Egyptian ophthalmy.

*Purulent Ophthalmia of Infants.*

Dr. Vetch's description of this disease is so accurate, that we shall give it in his own words.

"At first, the discharge is thin and whitish, afterwards, it becomes thicker and yellow; it collects in considerable quantities, and when the eyelids are forced open, it springs out with great force; occasionally it is mixed with blood. When the œdema ceases, the inner surface of the palpebræ becomes sarcomatous, and this diseased surface, when the eyelids are opened, forms an exterior fleshy circle, beyond which the relaxed conjunctiva of the eye comes forward as a second; and often the caruncula lachrymalis adds still further to the valvular appearance, which the part presents." P. 258.

*Treatment.* Leeches may be safely applied during the whole course of the complaint. When the tumefaction begins, a good effect will be experienced by inserting a small portion of an ointment composed of any animal fat, without wax, six drachms, and of hyd. nitrico-oxydum, ten grains. As the purulency advances, the liq. plumbi subacet. will be found as useful as in other instances of purulent ophthalmy. To assist the separation of the slough, a solution of the argenti nitras is serviceable. The recovery of the relaxed conjunctiva may be further aided by dropping, or injecting into the eye a solution of alumen, or of cupri sulphas.

We have seldom found any other application necessary, than the liq. zinci sulphatis injected with a syringe from the outer corner of the eye.

The plates accompanying this volume are not so well executed as we could have wished. We wish we could speak more favourably of the typographer. As, however, the work will be sure to pass through many editions, the printer will have an opportunity of recovering his credit, especially, if the author will take pains in carefully correcting the press.

We have seldom had an opportunity of perusing a book so replete with pathological science, and exhibiting such excellent and decided rules of practice, as the one before us. Dr. Vetch does not appear to write for the sake of spreading his name abroad, as is too much the fashion of the present day; but to establish facts, and to extend a correct knowledge of ophthalmic diseases, with the view of promoting civil and military hygiene. His close attention to the phœnomena of the purulent and gonorrhœal ophthalmia, is deserving of the highest praise; and his former appointment to the Military Ophthalmic Hospital, was a proof of merit, which we hope to see acknowledged with a more substantial reward. This treatise on the diseases of the eye should not only be read by the professed oculist, but most diligently studied by the physician, surgeon, and general practitioner.

